# Altitude and COPD prevalence: analysis of the PREPOCOL-PLATINO-BOLD-EPI-SCAN study

**DOI:** 10.1186/s12931-017-0643-5

**Published:** 2017-08-23

**Authors:** Andreas Horner, Joan B. Soriano, Milo A. Puhan, Michael Studnicka, Bernhard Kaiser, Lowie E. G. W. Vanfleteren, Louisa Gnatiuc, Peter Burney, Marc Miravitlles, Francisco García-Rio, Julio Ancochea, Ana M. Menezes, Rogelio Perez-Padilla, Maria Montes de Oca, Carlos A. Torres-Duque, Andres Caballero, Mauricio González-García, Sonia Buist, Maria Flamm, Bernd Lamprecht, for the BOLD Collaborative Research Group, the EPI-SCAN Team, the PLATINO Team, the PREPOCOL Study Group

**Affiliations:** 1 Department of Pulmonary Medicine, Kepler University Hospital, Krankenhausstrasse 9, A4021 Linz, Austria; 2 Faculty of Medicine, Johannes-Kepler-University, Linz, Austria; 3 Institute of General Practice, Family Medicine and Preventive Medicine, Paracelsus Medical University, Salzburg, Austria; 4 Instituto de Investigación Hospital Universitario de la Princesa (IISP), Universidad Autónoma de Madrid, Madrid, Spain; 5 Epidemiology, Biostatistics and Prevention Institute, University of Zurich, Zurich, Switzerland; 6 Department of Pulmonary Medicine, Paracelsus Medical University, Salzburg, Austria; 7 Department of Respiratory Medicine, Maastricht University Medical Centre, Maastricht, The Netherlands; 8 Program Development Centre, CIRO+, Centre of Expertise for Chronic Organ Failure, Horn, The Netherlands; 9 Respiratory Epidemiology and Public Health, Imperial College, London, UK; 10 Servicio de Neumología, Hospital Universitari Vall d’Hebron. Ciber de Enfermedades Respiratorias (CIBERES), Barcelona, Spain; 11 Servicio de Neumología, Hospital Universitario La Paz, IdiPAZ. Ciber de Enfermedades Respiratorias (CIBERES), Madrid, Spain; 12 Servicio de Neumología, Hospital La Princesa, Universidad Autónoma de Madrid, Madrid, Spain; 13 Programa de Pós-Graduacão em Epidemiologia, Universidade Federal de Pelotas, Pelotas, Brazil; 14 Institute of Respiratory Diseases, Instituto Nacional de Enfermedades Respiratorias, Mexico City, Mexico; 15 Servicio de Neumonología, Hospital Universitario de Caracas, Facultad de Medicina, Universidad Central de Venezuela, Caracas, Venezuela; 16 Departamento de Investigación, Fundación Neumológica Colombiana, Bogotá, Colombia; 17 Asociación Colombiana de Neumología y Cirugía de Tórax, Bogotá, Colombia; 18 Clínica Reina Sofía, Bogotá, Colombia; 19 Oregon Health and Science University, Portland, Oregon USA

**Keywords:** COPD, Geographical altitude, Risk factors, Underdiagnosis, Epidemiology

## Abstract

**Background:**

COPD prevalence is highly variable and geographical altitude has been linked to it, yet with conflicting results. We aimed to investigate this association, considering well known risk factors.

**Methods:**

A pooled analysis of individual data from the PREPOCOL-PLATINO-BOLD-EPI-SCAN studies was used to disentangle the population effect of geographical altitude on COPD prevalence. Post-bronchodilator FEV1/FVC below the lower limit of normal defined airflow limitation consistent with COPD. High altitude was defined as >1500 m above sea level. Undiagnosed COPD was considered when participants had airflow limitation but did not report a prior diagnosis of COPD.

**Results:**

Among 30,874 participants aged 56.1 ± 11.3 years from 44 sites worldwide, 55.8% were women, 49.6% never-smokers, and 12.9% (3978 subjects) were residing above 1500 m. COPD prevalence was significantly lower in participants living at high altitude with a prevalence of 8.5% compared to 9.9%, respectively (*p* < 0.005). However, known risk factors were significantly less frequent at high altitude. Hence, in the adjusted multivariate analysis, altitude itself had no significant influence on COPD prevalence. Living at high altitude, however, was associated with a significantly increased risk of undiagnosed COPD. Furthermore, subjects with airflow limitation living at high altitude reported significantly less respiratory symptoms compared to subjects residing at lower altitude.

**Conclusion:**

Living at high altitude is not associated with a difference in COPD prevalence after accounting for individual risk factors. However, high altitude itself was associated with an increased risk of undiagnosed COPD.

## Background

COPD is a common condition worldwide, but prevalence estimates are highly variable by time, geography, or other factors beyond age and smoking, which can only partly explain its population variability. Geographical altitude has been linked previously to COPD prevalence, yet with conflicting results [1–9]. First, the PLATINO study found that the greater the altitude, the lower the prevalence of COPD in five Latin American capitals [1]. In contrast, the PREPOCOL study, conducted in five Colombian cities, reported increasing COPD prevalence with higher altitude [2]. The influence of altitude on other medical conditions has been previously reported. For instance, reports consistently conclude that pulmonary hypertension and right heart failure have a higher prevalence in specific areas at high altitudes [10, 11]. On the other hand results from the Swiss National Cohort Study indicate that there is lower mortality from coronary heart disease and stroke at higher altitude [12, 13]. Tuberculosis incidence and mortality decreases with altitude, whereas mortality for pneumonia and influenza increases with altitude [14, 15].

Potential mechanisms behind the assumed impact of altitude on COPD prevalence are highly speculative including that altitude could induce a higher growth of airways relative to lung size, leading to an increased FEV_1_/FVC ratio. This may be an adaptation to external circumstances at high altitude such as chronic hypoxia, the necessary increase in resting ventilation or extreme physical performance, like in Sherpa populations [4, 16].

The PREPOCOL-PLATINO-BOLD-EPI-SCAN prevalence and underdiagnosis study was recently published [17]. This study pooled representative samples of adults aged 40 years and older randomly selected from well-defined administrative areas worldwide (44 sites from 27 countries), and has the potential to help to disentangle the population effect of geographical altitude on COPD prevalence. The primary objective of this analysis was to determine the association of COPD prevalence with altitude, taking into account well known COPD risk factors. Secondary objectives were to determine the association of COPD underdiagnosis with altitude and to determine the association of reported symptoms in participants with airflow limitation with altitude.

Some of the results of this study have been previously reported as an abstract [18].

## Methods

### Study populations

The PREPOCOL-PLATINO-BOLD-EPI-SCAN study methods have been described in detail elsewhere [17]. In summary, we used data from 30,874 participants enrolled in the following epidemiologic surveys: (1) BOLD, (2) PLATINO, (3) EPI-SCAN, and (4) PREPOCOL [1, 2, 19, 20].

The BOLD study [19, 21] is an ongoing population-based survey on COPD epidemiology. Field work for the data included was done from 2003 to 2012 and includes data from 16,218 men and women aged ≥40 years in 23 sites. Complete information, including questionnaire data and post-bronchodilator (post-BD) spirometry, were recorded. Details of the study protocol and prevalence of airway obstruction have been reported elsewhere [19, 21].

PLATINO was launched in 2002 in five Latin American cities in five countries. Complete information, including questionnaire data and post-BD spirometry, were recorded for 5315 participants aged ≥40 years [1, 22].

EPI-SCAN was a population-based survey conducted in 11 areas of Spain in 2007. Complete information, including questionnaire data and post-BD spirometry, were recorded for 3802 subjects aged ≥40 years [20, 23].

PREPOCOL was an urban population-based study conducted in five Colombian cities in 2003 to 2004. Questionnaire data and post-BD spirometry were recorded for 5539 subjects aged ≥40 years [2].

For all surveys, exclusion criteria were mental illness, institutionalization, inability to conduct spirometry, and contraindications to spirometry or salbutamol.

### Study measures

Post-BD spirometry after two puffs (200 μg) of salbutamol was performed in the four included studies. In both BOLD and PLATINO studies, spirometry was done according to American Thoracic Society (ATS) criteria [24] by trained and certified technicians using the ndd EasyOne spirometer (ndd Medical Technologies, Andover, MA, USA and Zurich, Switzerland). EPI-SCAN followed the same guidelines but used the MasterScope CT spirometer (VIASYS Health Care, Hoechberg, Germany). In PREPOCOL, spirometry was performed according to ATS criteria using the MicroLoop spirometer (Micro Medical Ltd., Rochester, Kent, UK). Quality control measures were done according to each study protocol [1, 2, 19–23]. In summary, all interviews and examinations were performed by certified staff, the spirometers were calibrated regularly and the spirometry results were reviewed centrally for quality by a third person. Moreover, regular feedback about the quality of their performance was given to each field worker during the period of data collection and retraining was undertaken as necessary.

### Questionnaire

The questionnaires used for the BOLD study [21], EPI-SCAN [23], PLATINO [22], and PREPOCOL [2] were administered by trained and certified staff in the participants’ native language and included information on respiratory symptoms, respiratory diagnoses, and risk factors for COPD. The questionnaires were translated from English into the study site language and then translated back to ensure accuracy.

### Definitions

COPD was defined by post-BD FEV_1_/FVC below the lower limit of normal (LLN) (persistent airflow limitation) and the Third National Health and Nutrition Examination Survey (NHANES) reference equations [25] were used to calculate predicted values. Ever smoking (current or former smoking) was defined as smoking >20 packs of cigarettes in a lifetime or more than one cigarette a day for 1 year. A prior lung function test was defined as present when the question, “Has a doctor or other health-care provider ever had you blow into a machine or device to measure your lungs?” was answered affirmatively. In case of doubt, the interviewers were able to explain the difference between a peak flow meter and a spirometer. A self-reported diagnosis of COPD, emphysema, or chronic bronchitis was based on questionnaire response (“Has a doctor or health-care provider ever told you that you have/had…?”). The reported diagnosis of COPD was considered correct if it was accompanied by post-BD airflow limitation (FEV_1_/FVC<LLN) at the time of the study visit. Undiagnosed COPD was considered when participants had post-BD FEV_1_/FVC<LLN but were not given a diagnosis of COPD by a physician or health-care professional. Severity of self-reported dyspnoea was recorded according to the modified Medical Research Council dyspnoea scale (0–4), with dyspnoea defined as present with a score ≥ 1. Presence of self-reported cough, phlegm, and wheezing was assessed using the following or similar questions: “Do you usually cough when you don’t have a cold?”; “Do you usually bring up phlegm from your chest, or do you usually have phlegm in your chest that is difficult to bring up when you don’t have a cold?”; “Have you ever had wheezing or whistling in your chest in the last 12 months?”.

Occupational exposure to dust or fumes was assessed by questionnaire. Additional measures evaluated included body mass index (kg/m^2^), total number of years of education, and self-reported physician-diagnosed comorbidities like tuberculosis or heart disease.

High altitude was defined as a geographical altitude of more than 1500 m above sea level. Although there is no generally acknowledged definition, in literature it is widely accepted as being the cut-off to influence the human body physiology [26–29].

The altitudes of all cities/sites were obtained from the original publications and, if not published, from elevationmap.net (http://www.elevationmap.net) in June 2016.

### Statistical analysis

**Fig. 1:**
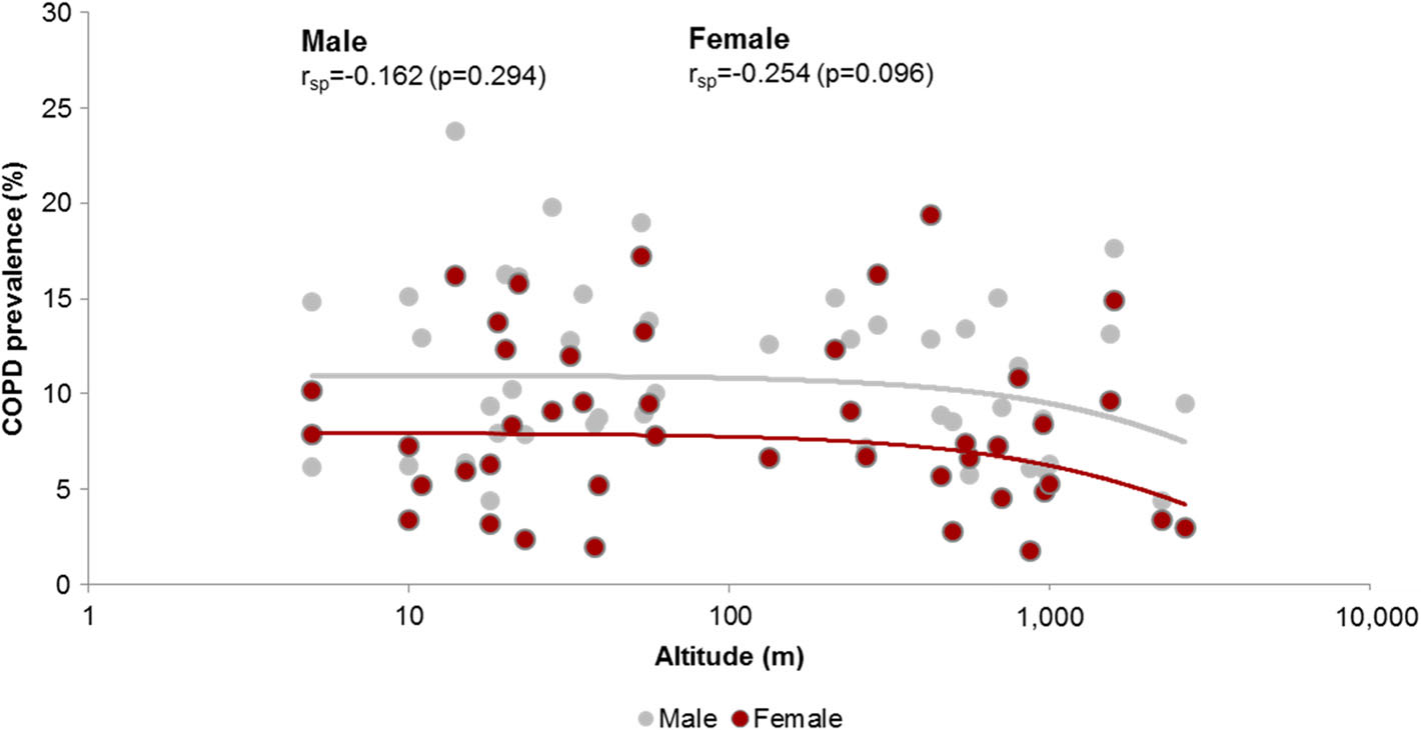
Scatterplot of the association of COPD prevalence by site (%) with altitude (m), with regression line explored by sex

**Fig. 2:**
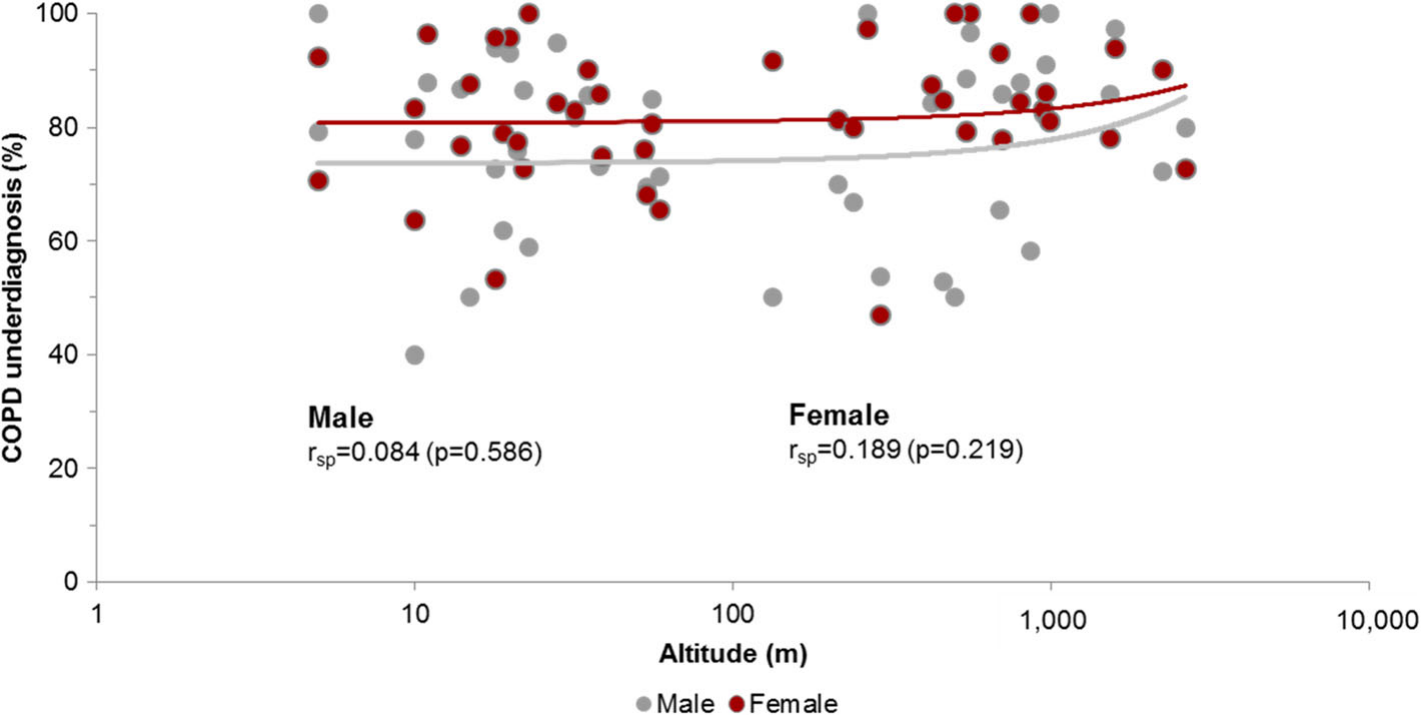
Scatterplot of the association of COPD underdiagnosis by site (%) with altitude (m) with regression line explored by sex

Data quality was centrally controlled, and a standardized template to translate all coding was applied. Variables were then double-checked by each principal investigator, and data considered as potential errors or outliers were individually discussed, and either confirmed or removed. Comprehensive tabulations with ranges, means, and SDs of all quantitative variables, and percentages of all qualitative variables were available for each study. All statistics were performed using R-3.3.0 (https://www.r-project.org). Results are expressed as mean ± SD for quantitative variables and count (percentage) for discrete variables. Parametric t-test, nonparametric Mann-Whitney U test, chi-square tests, and Spearman rank correlation coefficient were used to investigate differences, wherever appropriate. A Weibull model with logarithmic values for altitude was used for the scatterplot in Figs. 1 and 2.

A multivariate logistic regression model was used to examine the association of altitude with COPD prevalence while adjusting for sex, age, body mass index, level of education, smoking status, former tuberculosis, and a history of occupational exposure to dust.

Furthermore, a subgroup analysis in all participants with FEV_1_/FVC<LLN was performed to evaluate differences in reported symptoms at different altitudes.

In all analyses *p* < 0.05 was considered statistically significant.

## Results

Among 30,874 participants aged 56.1 ± 11.3 years from 44 sites worldwide, 55.8% were women, and 49.6% never-, 27.5% former- and 22.9% current-smokers. Overall, 12.9% or 3978 persons were residing at elevations above 1500 m, while the majority (51.3%) of the participants was living at altitudes less than 250 m above sea level. More detailed information on demographics, smoking status, geographical altitude and COPD prevalence by site is presented in Tables 1 and 2.

**Table 1:** Characteristics of participants

Characteristics		Total *n* = 30,874
Sex, n (%)	Female	17,230 (55.8)
	Male	13,644 (44.2)
Smoking status, n (%)	Never-smoker	15,308 (49.6)
	Former smoker	8479 (27.5)
	Current smoker	7065 (22.9)
	Missing information	22 (0.07)
Age in years, mean (± SD)		56.1 (±11.3)
Age in decades, n (%)	40–49	10,828 (35.1)
	50–59	9038 (29.3)
	60–69	6466 (20.9)
	70–79	3691 (12.0)
	≥80	851 (2.8)
Geographical altitude in meters, mean (± SD)		521.8 (±687.8)
Geographical altitude, n (%)	0–250	15,823 (51.3)
	250–500	3437 (11.1)
	500–750	2737 (8.9)
	750–1500	4899 (15.9)
	>1500	3978 (12.9)

**Table 2:** Altitude and prevalence of COPD (post-BD FEV1/FVC<LLN) by site and ascending order of altitude

	Site	Study	Altitude (m)	COPD prevalence (%)
1	Bergen, Norway	BOLD	5	12.5
2	Mumbai, India	BOLD	5	6.8
3	Barcelona, Spain	EPI-SCAN	10	10.7
4	Vigo, Spain	EPI-SCAN	10	4.8
5	Manila, Philippines	BOLD	11	8.5
6	CapeTown, South Africa	BOLD	14	19.0
7	Guangzhou, China	BOLD	18	7.8
8	Baranquilla, Colombia	Prepocol	18	3.6
9	Sydney, Australia	BOLD	19	10.9
10	Nampicuan, Philippines	BOLD	20	14.3
11	Uppsala, Sweden	BOLD	21	9.3
12	London, England	BOLD	22	16.0
13	Sevilla, Spain	EPI-SCAN	23	4.9
14	Adana, Turkey	BOLD	28	14.3
15	Vancouver, Canada	BOLD	32	12.3
16	Montevideo, Uruguay	Platino	35	11.9
17	Sousse, Tunisia	BOLD	38	5.0
18	Tartu, Estonia	BOLD	39	7.0
19	Maastricht, Netherlands	BOLD	53	18.2
20	Reykjavik, Iceland	BOLD	54	11.0
21	Lisbon, Portugal	BOLD	56	11.5
22	Hannover, Germany	BOLD	59	8.9
23	Cordoba, Spain	EPI-SCAN	133	9.4
24	Krakow, Poland	BOLD	214	13.7
25	Oviedo, Spain	EPI-SCAN	239	11.0
26	Ife, Nigeria	BOLD	266	6.9
27	Lexington, USA	BOLD	291	15.2
28	Salzburg, Austria	BOLD	424	15.8
29	Huesca, Spain	EPI-SCAN	457	7.2
30	Vic, Spain	EPI-SCAN	497	5.7
31	Santiago, Chile	Platino	543	9.7
32	Pune, India	BOLD	560	6.1
33	Requena (Valencia), Spain	EPI-SCAN	588	6.2
34	Madrid La Princesa, Spain	EPI-SCAN	648	10.9
35	Madrid La Paz, Spain	EPI-SCAN	648	6.6
36	Sao Paulo, Brazil	Platino	800	11.1
37	Burgos, Spain	EPI-SCAN	864	3.9
38	Caracas, Venezuela	Platino	950	8.5
39	Bucaramanga, Colombia	Prepocol	960	5.3
40	Cali, Colombia	Prepocol	995	5.6
41	Medellin, Colombia	Prepocol	1538	10.6
42	Srinagar, India	BOLD	1587	16.4
43	Mexico City, Mexico	Platino	2240	3.8
44	Bogotá, Colombia	Prepocol	2640	5.2

As shown in Table 3, COPD prevalence was significantly lower in participants living at high altitude defined as >1500 m above sea level, with a prevalence of 8.5% compared to 9.9%, respectively (*p* < 0.005). As seen in Fig. 1 this result was consistent for both men and women with Spearman’s rank correlation coefficients of −0.162 (*p* = 0.294) and −0.254 (*p* = 0.096), respectively. However, risk factors for airflow limitation such as smoking, a history of dusty work environment, or former tuberculosis were significantly less frequent at high altitude. Participants from high altitude were on average significantly younger and reported fewer years of education, see Table 3.

**Table 3:** Demographic characteristics and risk factors for COPD in subjects living at low (<1500 m) and high (>1500 m) altitude

Characteristics	Altitude <1500 m	Altitude >1500 m	*p*-value
	*n* = 26,896	*n* = 3978	
COPD prevalence (%)	9.9	8.5	<0.005
Mean FEV_1_ (Litre, SD)	2.66 (0,85)	2.45 (0.78)	0.010
Mean FVC (Litre, SD)	3.47 (1.05)	3.20 (0.90)	<0.001
Mean FEV_1_/FVC (SD)	76.8 (8.8)	76.2 (10.0)	<0.001
Sex – female (%)	54.9	62.1	<0.001
Age (Mean, SD)	56.2 (11.3)	55.1 (11.3)	<0.001
Never-smoker (%)	48.6	56.5	<0.001
Dusty job (%)	36.1	24.5	<0.001
Tuberculosis (%)^a^	3.5	0.4	<0.001
Education >12 years (%)	23.6	9.5	<0.001
Prior lung function test, ever (%)^b^	28.1	4.1	<0.001
Self-reported diagnosis of COPD (%)	5.1	3.9	<0.001
Proportion of correct prior diagnosis of COPD (%)	37.0	31.2	0.158
Proportion of undiagnosed COPD (%)	80.8	85.8	0.029

^a^14, 691 missing values

^b^5, 598 missing values

The results of the multivariate analysis adjusted for sex, age, body mass index, level of education, smoking status, and a history of occupational exposure to dust, as plotted in Table 4, do not show any significant association between living at high altitude (defined as >1500 m above sea level) and COPD prevalence.

**Table 4:** Crude and adjusted odds ratios for COPD (FEV1/FVC<LLN)

Variable		OR (crude) (95% CI)	*p*-value	OR (multivariate model) (95% CI)	*p*-value
Altitude	<1500	1		1	
	>1500	0.85 (0.75; 0.95)	0.005	0.90 (0.80; 1.02)	0.111
Sex	Male	1		1	
	Female	0.73 (0.68; 0.79)	<0.001	0.94 (0.86; 1.02)	0.119
Age in years		1.04 (1.04; 1.04)	<0.001	1.05 (1.04; 1.05)	<0.001
Years of education	>12	1		1	
	9–12	1.24 (1.11; 1.39)	<0.001	1.16 (1.03; 1.30)	0.015
	<9	1.48 (1.33; 1.64)	<0.001	1.24 (1.12; 1.39)	<0.001
Smoking status	Never	1		1	
	Former	1.97 (1.79; 2.16)	<0.001	1.78 (1.61; 1.97)	<0.001
	Current	2.78 (2.53; 3.05)	<0.001	3.40 (3.08; 3.76)	<0.001
Dusty job		1.37 (1.27; 1.48)	<0.001	1.18 (1.09; 1.29)	<0.001

Interestingly, people with airflow limitation (FEV_1_/FVC<LLN) living at high altitude are at significantly increased risk of not receiving a diagnosis of COPD (85.8%) compared to subjects at altitudes <1500 m (80.8%). This association was seen in both sexes with Spearman’s rank correlation coefficients of 0.084 (*p* = 0.586) for males and 0.189 (*p* = 0.219) for females (Fig. 2).

Only 4.1% of high altitude residents reported a previous lung function test, and only 3.9% stated a previous diagnosis of COPD compared to 28.1% and 5.1% of subjects living at altitudes <1500 m, respectively (Table 3). These differences were statistically significant. No significant difference was seen in the proportion of correct prior diagnosis of COPD.

**Fig. 3:**
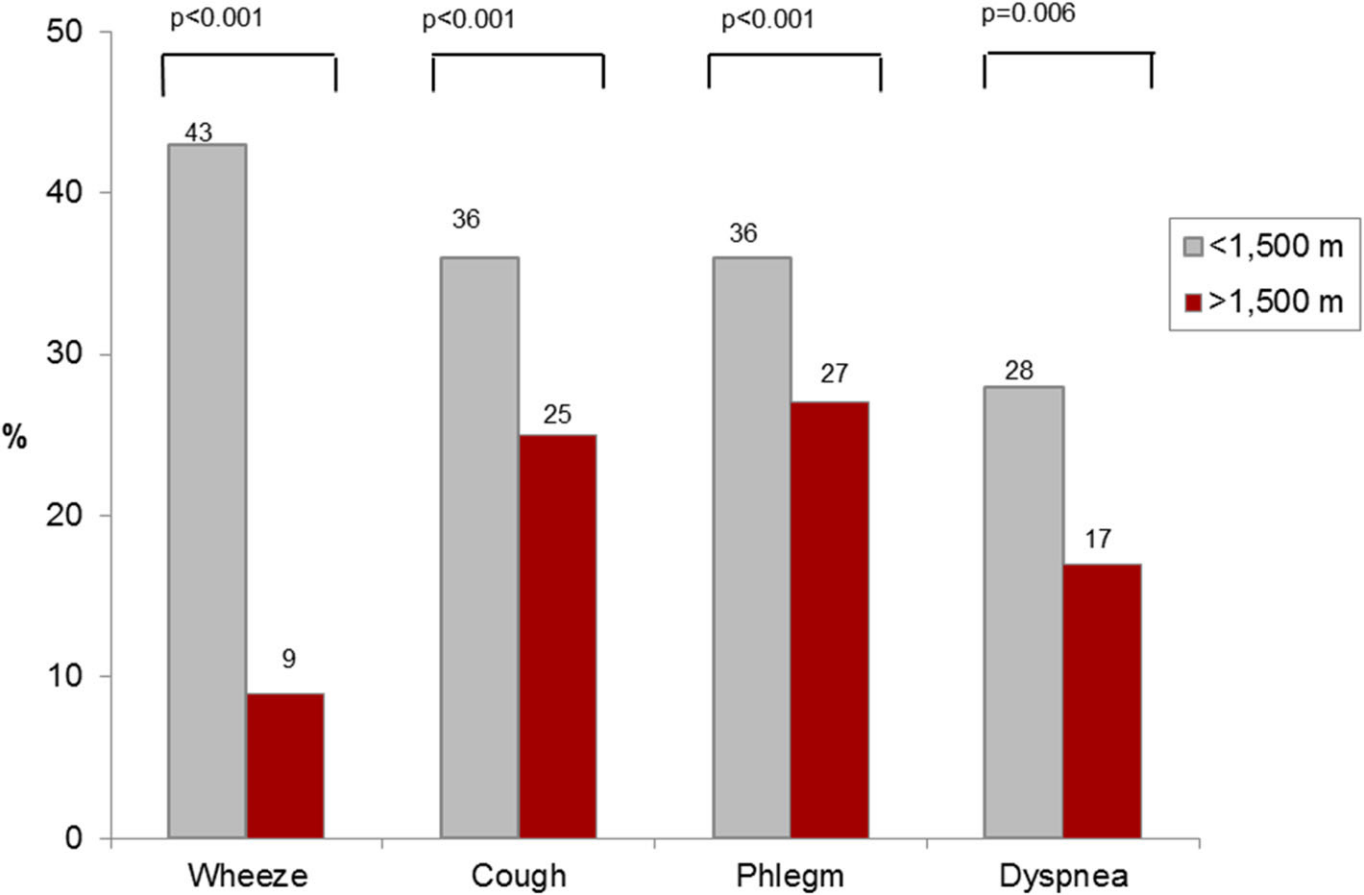
Self-reported respiratory symptoms by altitude in subjects with COPD (FEV1/FVC<LLN)

Figure 3 illustrates that participants living at high altitude, though having airflow limitation (FEV_1_/FVC<LLN), reported significantly less respiratory symptoms like cough, phlegm, dyspnoea or wheeze, compared to subjects residing at lower altitudes.

## Discussion

Our results indicate that living at high altitude is associated with a lower COPD prevalence. However, this could be considered an example of ecological fallacy, and in the multivariate analysis taking into account individual confounders, the association of altitude with COPD prevalence disappeared. Furthermore, living at high altitude was linked to an increased risk of undiagnosed COPD.

In the past, geographical altitude has been linked to COPD prevalence. Until now, the results concerning this topic were inconsistent. In some articles, an association between high altitude and higher COPD prevalence was mentioned, whereas other authors stated a possible protective effect of altitude for COPD.

PLATINO explored the association between altitude and COPD prevalence and reported that the prevalence was lowest in Mexico City (2240 m above sea level) and highest in Montevideo (35 m above sea level). There was a perfect correlation between the ranks of altitude in the five cities and the COPD prevalence rates (Spearman rank correlation coefficient − 1.0) [1]. It was noted that this finding was consistent with similar reports from the Himalayas [4, 16].

The PREPOCOL study reported opposite results [3]. In the general population without airflow limitation both FVC and FEV_1_ were significantly higher at higher altitude. However, FEV_1_ proportionally increased a little more than FVC, which could increase the FEV_1_/FVC ratio and decrease its ability to define the population with airflow limitation at a higher altitude. A non-significant tendency toward higher COPD prevalence with higher altitude was found, ranging from 6.2% in Barranquilla (18 m above sea level) to 13.5% in Medellín (1538 m above sea level) [2].

A recent study conducted in four Peruvian regions reported that COPD prevalence was highest at the highest altitude, with a RR = 1.6 versus sea level [5], therefore being more consistent with PREPOCOL.

Laniado et al. [6] reported a COPD case-finding study that included 27 Mexican cities, within an altitude range from 1 to 2680 m above sea level. They described a weak (−0.31; *p* < 0.0001) although significant negative correlation between altitude and COPD prevalence. The COPD rate for cities located ≤1000 m was 32.7% vs 16.4% for cities located >1000 m (*p* < 0.0001); the rate for cities located at ≤2000 m was 22.7% vs 15.6% for those >2000 m.

A recent study by Chan et al. analysed the geographic disparity in mortality rates in Taiwan and found a statistically significant inverse relationship between COPD mortality and altitude [7].

A recent meta-analysis by Aaron et al. of 80 articles published during 2003–2014 found a protective effect of high elevation above sea level for COPD [9]. However, risk factors were only analysed on country-level and not on an individual subject level.

Our study has a number of strengths that help to disentangle these conflicting results. To our knowledge, it is the largest analysis (*n* > 30,000) of the effect of altitude on COPD prevalence with a multinational approach, including various ethnicities and races. Both spirometry and bronchodilator testing were conducted with similar protocols, which likely reduces bias due to methodologic issues. By using the LLN, we avoided problems associated with the use of the fixed ratio of FEV_1_/FVC, allowing us to better compare results across studies [30].

A high prevalence of spirometric restriction is likely to occur in low-income countries, and low FVC is likely related to poverty, low birth weight, poor diet, early infections, and exposure to indoor air pollution (burning of biomass fuel) [31–36]. Because local prediction equations may underestimate the local severity, and to be consistent with earlier BOLD publications we deliberately used the LLN for height, age, and sex on the basis of the Third National Health and Nutrition Examination Survey reference population [25].

COPD mortality is associated with low vital capacity [37]. As the prognostic significance of a given FVC is independent of ethnicity [38], we decided not to adjust the LLN for ethnicity.

High rates of undiagnosed COPD at high altitude may be influenced by the fact that these subjects tend to report significantly less respiratory symptoms. This result is consistent with a previous analysis of our group where COPD underdiagnosis was associated with a lack of reported respiratory symptoms, no previous spirometry and lower education amongst others [17]. However, to reduce underdiagnosis is relevant for COPD patients considering that Çolak et al. reported recently that individuals with undiagnosed symptomatic COPD have an increased risk of exacerbations, pneumonia, and death. Additionally, even individuals with asymptomatic undiagnosed COPD had an increased risk of exacerbations and pneumonia [39, 40].

Furthermore, there is only a poor correlation between reported COPD symptoms and lung function (FEV_1_), and reported symptoms vary in different studies [41]. Hence, other circumstances that come with living at high altitude such as greater poverty, poorer education, lower health literacy, and lack of access to health care resources, may play a role in underdiagnosis of COPD.

Although the proportion of subjects with a previous lung function test is far higher at low altitude, this did not influence the proportion of correct diagnoses of COPD.

However, some limitations must be noted. All four surveys used different tools and brands of spirometers with somewhat different protocols and quality control. Nevertheless, all spirometers were calibrated and set up to the same ATS/European Respiratory Society guidelines. International variation was also seen across the 23 BOLD sites using the same spirometer (ndd EasyOne), standardized protocol and quality control. Countries assessed are in different stages of the tobacco epidemic, perhaps even by region and other risk factors have a variable distribution, for instance biomass fuel, occupational exposure, educational and poverty levels. However, the PLATINO study has shown that underdiagnosis is as common in subjects with airflow limitation who never smoked as in ever-smokers [42].

Although we adjusted for education as an indicator of socioeconomic status in our multivariate model, there may be other factors that influence COPD prevalence that we were not able to assess. For example, it has been shown, that Hispanic ethnicity is inversely associated with spirometric COPD prevalence even after adjustment for smoking [43]. Occupational exposure to dust was assessed using different questionnaires for workplace exposures in the original studies. Hence, a recall bias may be possible concerning this risk factor.

Some additional determinants of COPD prevalence for example indoor and outdoor air pollution, and biomass exposure [44–46] were not assessed in all the included studies and therefore not analysed. Hence, further research is needed regarding these parameters. Additionally, as a decreasing trend in COPD prevalence in very high altitude cities (> 2000 m above sea level) was seen, further research in the field of COPD prevalence at very high altitude is desirable.

Although the proportion of missing information concerning most parameters was negligible, there was no data available about former tuberculosis in 14,691 subjects and in 5598 individuals about a prior lung function test. Altitude of all study sites was taken from only one geographic point, the central agglomeration where the study centre was located. These study sites had different catchment areas wherein the altitude of the actual residence of the subjects may vary. However, as the main results of our analysis are based on differences between low and high altitude (>1500 m above sea level), minor differences in altitude should not influence the results. The effect of birth place, seasonal traveling and other individual movements, should produce non-differential effects. Unfortunately, we do not have any information if study subjects lived on the same altitude during their whole life or have moved up or down. This may affect disease prevalence as described by Faeh et al. [12] in their Swiss study for coronary heart disease and stroke.

## Conclusions

Living at high altitude is associated with a lower COPD prevalence, but it seems that other related individual risk factors are accountable for this finding. After adjustment for confounders there is no association between high altitude and COPD prevalence. However, high altitude itself is associated with an increased risk of undiagnosed COPD. This may be caused by the fact that individuals at high altitude were less symptomatic despite having an obstructive pattern in their lung function test. Further research is needed to evaluate other risk factors like biomass exposure, indoor and outdoor air pollution, and poverty.

## Abbreviations

ATS: American Thoracic Society
BOLD: Burden of Obstructive Lung Disease
COPD: Chronic Obstructive Pulmonary Disease
EPI-SCAN: Epidemiologic Study of COPD in Spain
LLN: Lower limit of normal
NHANES: Third National Health and Nutrition Examination Survey
PLATINO: The Latin American Project of the Investigation of Obstructive Lung Diesease
post-BD: Post-bronchodilator
PREPOCOL: Prevalence Study of COPD in Colombia
